# The quality of Syrian healthcare services during COVID-19: A HEALTHQUAL approach

**DOI:** 10.3389/fpubh.2022.970922

**Published:** 2022-08-11

**Authors:** Lilas Allahham, Sulaiman Mouselli, Mihajlo Jakovljevic

**Affiliations:** ^1^Faculty of Business Administration, Arab International University, Daraa, Syria; ^2^Institute of Advanced Manufacturing Technologies Peter the Great St. Petersburg Polytechnic University, Saint Petersburg, Russia; ^3^Institute of Comparative Economic Studies, Hosei University, Chiyoda, Japan; ^4^Department of Global Health Economics and Policy, University of Kragujevac, Kragujevac, Serbia

**Keywords:** healthcare service quality, public hospitals, private hospitals, COVID-19, HEALTHQUAL, Syria

## Abstract

Syria is a developing country that face enormous healthcare challenges that aggravated with the outbreak of COVID-19. In the study, we evaluate the perceived healthcare service quality based on hospital type, public and private, using five HEALTHQUAL dimensions. We find that service quality in Syrian private hospitals is perceived better that in public hospitals. However, neither type of hospitals scores exceptionally high in any of the examined HEALTHQUAL dimensions. On the contrary, both hospitals score extremely low in the Improvement dimension. We argue that crowdedness environment, medical staff availability and their low salaries, pricing policies as well as the health insurance system, are to blame for such low perceived quality.

## Introduction

The Syrian healthcare system has a complex nature and has long been subject to changes amid political and economic conditions. Public hospitals were the backbone of the Syrian healthcare system pre-war and largely belong to the Ministry of Health, Ministry of Higher Education and Scientific Research and Ministry of Defense. The combined impact of wartime destruction, healthcare worker migration, poor working conditions, and severe budgetary shortfalls led to the deterioration of public hospitals' services and allowed private hospitals to increasingly compensated for poorly public services (1). However, public hospitals remain the main provider of free or cheap medications for chronic diseases (2).

The Syrian economic regime has adopted socialism since 1963. However, the Syrian government started to open the economy since 1980s and encouraged the participation of private sector. However, it was until 2005 when the 10^th^ national conference of Al-Baath party officially announced the move to social market economy. Such move reduced the government intervention in economic activities and opened the door for private sector to participate in economic activities and healthcare is no exception. Expectedly, the government expenditure on health as percentage of GDP declined from 4.9 percent in 2005 to 3.4 percent in 2010 (3). Such decline was enormous if we consider the increase in prices and population and it leads to increase out-of-pocket spending on healthcare to compensate for the declining role of public healthcare services. In addition, some public hospitals started to offer paid services for those who are better off with noticeable differences in service quality between both types of patients (4).

Further reductions in government spending on health are recorder since 2010. According to recent projected budgetary figures, the Syrian government expenditure on health has declined in 2020, in real terms, to reach less than half of 2011 figures (5). This situation represents a decline in health expenditure per capita from $9.72 in 2011 to $4.49 in 2020 (5). In 2020, there was 507 hospitals, disproportionally distributed between cities and among public and private hospitals, with 114 public and 393 private hospitals and an average number of persons per bed of 706 (5) which is lower than the average number of persons per bed in 2011 of 734 (6).

The Syrian healthcare system has long been based on out-of-pocket payments, which represents 53.69 percent of health expenditure in 2012 (7). Health insurance has gained grounds among public workers after a national legislation that made health insurance as compulsory for all public workers. The majority of workers in the private sector are health insured as part of their salary package. However, self-employed workers, such as farmers, remain out of the health insurance coverage. The percentage of health insured persons is <5 percent of the whole population in 2020 (8). Yet, this health insurance is far from being universal and is poorly administrated which force well-off patients to give up the service and pay for their own treatment. Uncovered patients still prefer to visit public hospitals which are always open to all.

Private hospitals have been considered as logical alternatives to public hospitals and are expected to relieve some burden from public hospitals. However, the underregulated and profit-driven incentives private hospitals diminished the utility of private services in responding to COVID-19 (1). In addition, private hospitals suffered from similar shortages and problems as government hospitals (9).

Syria has witnessed a significant daily increase in the number of COVID-19 cases and an increase in mortality rates among patients, medical and nursing staff (10). In addition, it faced a shortage of many basic tools and equipment to treat patients, the most important of which are oxygen tubes. Many non-government initiatives were launched to support the government efforts and overcome these challenges.

Overcrowding in Syrian public hospitals is not a recent result of the pandemic. During the Syrian war, many areas were destroyed, and the migration of large numbers of people to safer areas increases, including the capital, Damascus. This displacement led to an increase in patient's volume in public hospitals. Private hospitals, motivated by the aim of continuing their usual surgeries, refrained from accommodating COVID-19 positive patients. These patients were prescribed medications and sent home quickly with all necessary instructions.

This gloomy image of the Syrian healthcare means that COVID-19 pandemic comes to Syria in a very difficult time to add insult to injury. Despite the strict measures imposed by the Syrian government in the form of distancing and precautionary measures, the lack of ventilators and personal protective equipment (PPE), in public and private hospitals, are thought to have resulted in a troubling number of deaths among patients and healthcare worker alike (1). Patients become more reluctant to visit hospitals during the pandemic. Public hospitals were viewed as “less as treatment centers and more as potential sites of transmission” (1). Moreover, insurance companies claimed that health insurance policies do not cover COVID-19 treatment. Furthermore, private hospitals refused to admit COVID-19 patients, and the cost of treatment in hospitals that admit COVID-19 patients was prohibitively high for the average Syrian. The end result of this situation is that Covid-19 patients had to choose home treatment and the quality of care and patient safety, whether of COVID-19 patients or others, were extremely questionable.

## Importance of research

The focus of policymakers usually shifts during pandemics from the quality of care and safety of patients *per se* to the management of the pandemic itself. However, providing quality care and making things safe for patients will be more challenging during pandemics. Out of fear, lockdown restrictions, or insufficient availability of staff and resources at health facilities, many Syrian patients, including COVID-19 positive cases, refrained from visiting emergency departments, delayed operations, or missed their scheduled check-up. While healthcare systems in many countries have prioritized COVID-19 patients, the opposite was true in Syria and COVID-19 patients have failed to receive the appropriate care.

The low quality of health services has severe direct and indirect consequences. In addition to the loss of an organization's customers, if the organization's services are not of good quality, poor healthcare services will have a tremendous impact on the spread of the virus (11). Yet, public hospitals are unconcerned with such customer loss given that they are centrally funded and not profit-driven. A systematic analysis concluded that poor healthcare quality was the primary factor contributing to an increase in fatalities from cardiovascular disease, newborn traumas, and communicable diseases (12). Patients are not the only victims of low healthcare quality, but also the worsening quality of healthcare provided makes doctors more prone to workplace violence. Mohamad et al. (13) reported that 84.74 percent of resident doctors at public hospitals exposed to verbal violence while 19.08 percent exposed to physical violence.

COVID-19 related research in Syria is relatively rare and focused on Syrians' knowledge of the disease. Al Ahdab (9) found that Syrian residents demonstrated modest knowledge, attitudes and practices toward COVID-19 during the pandemic. Shibani et al. (14) confirmed the knowledge gap regarding many aspects of the disease and the hesitancy of Syrians toward taking the COVID-19 vaccines. This research endeavors to test the quality of Syrian healthcare system in the very difficult time of COVID-19 pandemic outbreak using HEALTHQUAL scale. It will also compare the quality of healthcare services between public and private hospitals according to the five dimensions of HEALTHQUAL scale.

## Literature review

The attempts to evaluate the service quality in the healthcare sector were old and enormous and can be traced to Donabedian (15), who discussed the pros and cons of a number of methods and approaches used to evaluate the medical care quality. Myers (16) considered accessibility, effectiveness, efficiency, and improvement of care quality and continuity as items for healthcare safety. Donabedian (17) added equity and efficiency as additional items to quality measurement that are related to patient care experience. Grönroos (18) developed the first service quality model to measure service quality based on qualitative methods. Then, Parasuraman et al. (19) developed the second service quality model (SERVQUAL) on the basis of exploratory research, in which service quality is seen as a function of the differences between customer expectations and service performance. SERVQUAL was based on five dimensions: tangibles, reliability, responsiveness, assurance, and empathy. Cronin and Taylor (20) proposed the weighed service performance (SERVPERF) model. SERVPERF was based on the five dimensions of SERVQUAL and 22 items to measure service quality but did not use the gap between expectations and service performance. Jain and Gupta (21) argued that SERVPERF framework was mythologically an improvement over SERVQUAL.

A number of studies attempted to add, reduce or change the SERVQUAL dimensions to accommodate different settings such as Carmen (22), Bowers et al. (23), Jun et al. (24), Shelton (25), Doran and Smith (26), Mostafa (27), Scobie et al. (28), Evans and Lindsay (29), Yesilada and Direktor (30). Rahim et al. (31) used machine learning to build a sentiment analyzer and service quality classifier, instead of questionnaire, to automatically classifies the sentiment and SERVQUAL dimensions using comments from 48 official public hospitals' Facebook pages.

Lee (32) proposed HEALTHQUAL as a measurement of healthcare service quality on the basis of tangibility, efficiency, safety, empathy, and improvements of care services. HEALTHQUAL is a multidimensional scale that combines patient's view with hospital view while considering the perspective of accreditation institutions. Such patient-centered perspective is largely influenced by a cultural milleu and has some common shared features across vast geography of Arabic League or MENA countries (33).

There have been several attempts to compare service quality in public vs. private hospitals before the spread of COVID-19. Andaleeb (34) argue that private hospitals were more motivated than public hospitals to offer higher service quality since these hospitals depend on income from patients. Many researchers supported this view in their findings regarding patients' perceptions of private and public hospitals' service quality (35–42). However, other studies argued that the reverse is true (39, 43, 44). Rahim et al. (31) founnd that patients in Malaysia were generally satisfied with the services provided by public hospitals though they did not compare with private hospitals.

Studies on the quality of healthcare in Syria is sporadic. Alfarraj (45) and Mahmoud (46) examined the quality of the healthcare merely in public hospitals, i.e., in the Ministry of Higher Education and the Ministry of Health, respectively. Such examinations were carried out in war-free, pandemic-free periods and did not compare healthcare quality between public and private hospitals. In addition, both studies considered limited dimensions of healthcare quality and concluded that patients positively perceived the quality of healthcare service at public hospitals. Despite the frequent adaptations of the HEALTHQUAL survey to measure perceived satisfaction, to date, no studies have been conducted using the HEALTHQUAL scale in Syria.

## Methods

In this study, we analyze the quality of healthcare service using five dimensions HEALTHQUAL adapted from and Kim (47). Thus, our HEALTHQUAL scale compromises of five constructs and a total of 27 items: (1) satisfaction with facilities and equipment (6 items); (2) satisfaction with safety (5 items); (3) perceived empathy (7 items); (4) perceived efficiency (5 items); (5) perceived improvements of care service (4 items).

A descriptive, exploratory, cross-sectional study was carried out during 2021. An internet-based questionnaire on the basis of the above-described HEALTHQUAL scale was applied to a sample of 220 visitors to public and private hospitals during the COVID-19 pandemic outbreak. All items were measured on a 5-point Likert scale, where five was “strongly agree” and one was “strongly disagree.” Respondents to the questionnaire were informed that the data collection was anonymous and the purpose of this research is only of scientific objectives.

Table 1 illustrates the demographic characteristics of the respondents according to hospital type, public or private. It shows that there were 152 respondents that have visited private hospitals compared to only 68 who went to public hospitals. In addition, the main age group in our sample is the smallest one (the age range of 18 to 34 years) with 115 respondents. It also shows that females dominate our sample with 130 respondents.

**Table 1:** Distribution of the surveyed visitors according to age, gender, and hospital type.

**Age**	**Public hospital**		**Private hospital**		**Total**
	**Male**	**Female**	**Male**	**Female**	
18–34	31	14	19	51	115
35–54	7	11	16	25	59
+55	2	3	15	26	46
Total	40	28	50	102	220

## Results

Table 2 shows the results from the combined sample of visitors to both public and private hospitals. The means of respondents' scores on Readiness, Safety, Empathy, and Efficiency range between 2.56 and 3.36. However, there is a serious issue regarding the mean scores of improvement items: appropriateness of care service provided (1.13), degree of improved patient condition after using this hospital care (1.26) and complete and comprehensive health services in the hospital (and is referred to other specialists if necessary) (1.27). These scores indicate that the Syrian healthcare services has serious problems with the improvement dimension of HELATHQUAL.

**Table 2:** Measurement items of HEALTHQUAL.

**Construct/Indicator**	**Code**	**Mean**	**SD**
**Readiness (Tangibles)**			
- Degree of securing advanced medical equipment	R1	2.97	1.251
- Degree of securing medical staff with advanced skills and knowledge	R2	3.20	1.203
- Degree of convenient facilities	R3	2.69	1.313
- Degree of continuous hygiene and sterilization	R4	3.10	1.299
- Degree of cleanliness of employee uniforms	R5	3.18	1.269
- Overall cleanliness of the hospital	R6	3.28	1.283
**Safety**			
- Degree of a comfortable and safe environment for receiving treatment	S1	3.17	1.187
- Degree of the feeling that doctors would not make misdiagnoses	S2	3.11	1.307
- Degree of the feeling that nurses would not make mistakes	S3	3.01	1.253
- Degree of confidence about the medical proficiency of this hospital	S4	3.14	1.218
- Degree of a hospital environment that is safe from infection	S5	2.90	1.407
**Improvement**			
- Appropriateness of care service provided	Q1	1.13	0.729
- Recognition and efforts for the best treatment by the medical staff	Q2	3.07	1.152
-Degree of improved patient condition after using this hospital care	Q3	1.26	0.656
-Complete and comprehensive health services in the hospital (and is referred to other specialists if necessary)	Q4	1.27	0.744
**Efficiency**			
- Attitudes about not using unnecessary medication	F1	3.07	1.383
-Providing patient the side effects of medication	F2	2.56	1.318
- Degree of efforts for providing appropriate treatment methods	F3	3.23	1.196
- Degree of convenience for treatment procedures	F4	3.09	1.226
- Degree of efforts for reducing unnecessary procedures	F5	3.04	1.267
**Empathy**			
-Polite attitudes of employees	E1	3.24	1.162
-Explaining the details	E2	3.24	1.213
-Listen to the patient	E3	3.20	1.183
-Understand and consider the patient's situation	E4	3.36	1.273
-A sense of closeness and friendliness	E5	3.10	1.242
-Hospital knows what the patient wants (meet their needs).	E6	2.99	1.235
-Hospital understands the patient's problems as empathy	E7	3.00	1.259

Table 3 presents the five constructs of HEALTHQUAL together with their 27 items. To illustrate the individual viability of each item, the factor loadings and composite reliability for each construct are also reported. As can be seen, the factor loadings obtained from Principal Component Analysis in most of the indicators were > 0.70, demonstrating that the proposed indicators are suitable for the constructs. Eigen values for Readiness, Safety, Improvement, Efficiency, and Empathy are 4.279, 3.930, 2.868, 3.110, and 5.468, respectively. The percentage of variance explained are Readiness (71.31), Safety (78.594), Improvement (67.162), Efficiency (62.196), and Empathy (78.11).

**Table 3:** Factor loadings and composite reliability of HEALTHQUAL.

**Construct/Indicator**	**PCA**			**Composite** **reliability: ** **Chronbach's** **alpha**
	**Factor** **loadings**	**Total** **eigen** **values**	**% of** **variance** **explained**	
**Readiness (Tangibles)**		4.279	71.310	0.919
R1	0.826			
R2	0.761			
R3	0.85			
R4	0.872			
R5	0.868			
R6	0.884			
**Safety**		3.930	78.594	0.930
S1	0.876			
S2	0.893			
S3	0.891			
S4	0.931			
S5	0.839			
**Improvement**		2.686	67.162	0.818
Q1	0.853			
Q2	0.855			
Q3	0.792			
Q4	0.775			
**Efficiency**		3.110	62.196	0.846
F1	0.832			
F2	0.792			
F3	0.846			
F4	0.712			
F5	0.754			
**Empathy**		5.468	78.110	0.953
E1	0.861			
E2	0.831			
E3	0.907			
E4	0.87			
E5	0.905			
E6	0.906			
E7	0.904			

Reliability was tested on the basis of Cronbach's alpha values (Table 3). All of the coefficients of reliability for the constructs exceeded the threshold value of 0.70 for exploratory constructs. In the reliability test, the Cronbach's alpha value for empathy was the highest with 0.953 and improvement was the lowest, 0.818.

Table 4 illustrates the descriptive statistics of HEALTHQUAL dimensions according to hospital type. Private hospitals scored higher than public hospitals at all dimensions which indicates better service quality at private hospitals in comparison to public hospitals during COVID-19 spread, which is consistent with (48). The *t*-test for the equality of means suggests that private hospitals superiority is significant at five percent level of significance. Surprisingly, both hospitals score low at improvement dimension but private hospitals still outperforming public hospitals in this regard. In general, the results show that private hospitals surpassed public hospitals by achieving high rates in all dimensions of HEALTHQUAL.

**Table 4:** Descriptive Statistics of HEALTHQUAL dimensions according to the type of hospital (public vs. private).

**Hospital type**		***N***	**Mean**	**Std. deviation**	**Std. error mean**	***t*-test for equality of means**
Readiness	Public	68	2.4265	0.95260	0.11552	−6.485
	Private	152	3.3575	0.99768	0.08092	
Safety	Public	68	2.5618	1.10859	0.13444	−4.641
	Private	152	3.2921	1.06501	0.08638	
Improvement	Public	68	1.3750	0.63113	0.07654	−4.703
	Private	152	1.8191	0.65433	0.05307	
Efficiency	Public	68	2.7412	0.94383	0.11446	−2.554
	Private	152	3.1118	1.01635	0.08244	
Empathy	Public	68	2.7059	1.02270	0.12402	−4.356
	Private	152	3.3637	1.04071	0.08441	

Regarding readiness, we found a statistically significant difference in respondents' evaluation of readiness between public and private hospitals in favor of the private hospitals. The mean of perceived readiness for private hospitals is (3.35 ± 0.997) is higher the mean of responses regarding the readiness of public hospitals (2.42 ± 0.952). Moreover, the difference in perceived readiness is in favor of private hospitals and is statistically significant with t-statistics of −6.485. This result can be explained by funding shortages due to war conditions that reduced the availability of necessary facilities and hygiene issues. In addition, personnel at public hospitals did not pay enough attention to hygiene issues due to the low self-awareness toward sterilization and personal hygiene guidelines (9, 49, 50).

Private hospitals have modernly designed buildings and attractive rooms, in addition of equipment and medical tools that surpass public hospitals. Private hospitals can easily adjust their prices to provide the necessary facilities and to cover the purchase of necessary hygiene equipment and to hire skilled staff. These results are attributed to several reasons, the most important of which is that private hospitals have modernly designed buildings and attractive rooms, in addition to medical equipment, tools and equipment that exceed public hospitals, whose buildings are old and neglected and in need of modification. Hospitals must provide a sophisticated and safe treatment environment for patients and staff that enhances a sense of comfort and safety.

The results from the Safety dimension illustrate a mean of (3.29 ± 0.1.06) against the mean of responses in public sector (2.65 ± 1.108) and the difference is statistically significant at five percent level of significance. Syrian patients feel more comfortable and safer while treated at private hospitals compared to public hospitals possibly because they are less-crowded than public hospitals. Crowded environment stands as a major obstacle in improving the service quality in public hospitals. Another reason for these differences is related to the pricing policies where public hospitals treatment costs are free or symbolic and the income of medical staff at public hospitals is low and makes them careless in terms of diagnosis and follow up. Previous studies show that patients with high income receive better healthcare service (35, 51–53).

The main purpose of improvement dimension is to measure whether the medical services meet the needs of patients and whether the patient feels satisfied during and after providing the services. Sharifi (54) called this dimension “effectiveness” which is related to patient's goals in receiving the appropriate and complete treatment from the hospital. The results in Table 4 above show the dissatisfaction of respondents from this dimension from both hospitals with a mean of (1.81 ± 0.65) for the private hospitals in comparison to (1.37 ± 0.63) for the public hospitals. We conclude that the services provided in both public and private hospitals during the pandemic were unable to meet the requirements and needs of patients and that they did not feel that their health conditions improved after using the healthcare service. This is despite that private hospitals scored significantly higher than public hospitals on this dimension.

In terms of efficiency, private hospitals score higher than public hospitals in this dimension with an averages of (3.11 ± 1.016) and (2.74 ± 0.94), respectively. Moreover, this difference is statistically significant at five percent level of significance. However, this dimension has the lowest difference between public and private sector. This can be partially explained by the fact that public hospitals still attract expert medical staff who are highly experienced doctors. Those medical staff are still working in the public sector despite their low salaries either because they have contractual obligations or because they use their positions at public hospitals as tool to provide their private patients easy access to cheap public healthcare services.

The results from the empathy dimension confirms previous dimension results. That is, private hospitals outperform public hospitals in terms of perceived empathy with averages of (3.36 ± 1.04) and (2.705 ± 1.02) for private and public hospitals, respectively. Again, the difference between averages is in favor of private hospitals and is statistically significant at the five percent level of significance. The overcrowded environment at public hospitals and shortages in medical staff do not permit medical staff spend enough time with patients and develop the sense of closeness and friendship. That is medical staff are forced, sometimes, to work beyond their knowledge and expertise to fill the shortage of services gap (55), and have less time to build rapport with patients, deteriorating the doctor–patient relationship. On the contrary, medical staff at private hospitals are in a better position to listen to patients and explain every detail of their treatment. In addition, they are well-paid and care about patients' feedback and satisfaction from their services.

In order to investigate which of the examined variables affect the improvement dimension, we run the following linear regression for each type of hospitals separately,


$$\begin{array}{cc} \text{Improvemen}{\text{t}}_{\text{i}}=\text{α}+{\text{β}}_{1}\text{Readines}{\text{s}}_{\text{i}}+{\text{β}}_{2}{\text{Safety}}_{\text{i}}+{\text{β}}_{3}\text{Efficienc}{\text{y}}_{\text{i}}\\ + & {\text{β}}_{4}\text{Empath}{\text{y}}_{\text{i}}+{\text{ε}}_{\text{i}} \end{array}$$

The results from estimating the above equation can be seen in Table 5 below. It can be seen that readiness is a significant determinant of public hospitals improvement dimension with a coefficient of 0.206 that is significant at one percent level of significance. Readiness is the most important factor in this analysis with a standardized coefficient of 0.311. Safety and Empathy are only significant at 10 percent level of significance while efficiency is insignificant at all levels. The insignificant impact of efficiency on Improvement is due to the fact that public hospitals have well trained medical staff. Yet, these hospitals failed to meet patients' needs and ambitious as patients did not feel better after using their healthcare service due to overcrowding and resource shortages.

**Table 5:** The Regression of improvement on independent variables.

**Variable**	**Public hospitals**		**Private hospitals**	
	**β**	**Standardized coefficients**	**β**	**Standardized coefficients**
α	−0.112		−0.127	
Readiness	0.206^***^	0.311	0.169^***^	0.257
Safety	0.152^*^	0.268	0.104^*^	0.169
Efficiency	0.083	0.124	0.225^**^	0.349
Empathy	0.136^*^	0.221	0.101^**^	0.161
Adj-*R*^2^	0.681		0.689	
F-Statistic	36.777		84.747	
*P*-value	0.000		0.000	

***,**,* represent significance at 1, 5, and 10 percent, respectively.

When estimating the same equation on private hospitals, we also find that readiness is a significant determinant of improvement at one percent level of significance and efficiency is also significant and has the highest standardized coefficients with 0.349. This suggests that patients give more importance to efficiency than readiness as determinant factor of improvement. Empathy has also a positive and significant impact on improvement with a coefficient of 0.101. In addition, Safety has a positive and significant impact on improvement but only at 10 percent level of significance.

To address the problem of endogeneity and as part of the robustness tests, we investigate if the variable Efficiency plays a mediator role in the relationship between the other three variables, Readiness, Safety, and Empathy, and Improvement. Thus, we construct a variable (RSE) as the average of these three variables and disentangle the direct and indirect effects of these variables on Improvement. The results from the public hospitals analysis in Table 6 indicate that the effect of the above-mentioned three variables on Improvement is predominantly direct with a coefficient of 0.164 of the total effect of 0.184. The results from private hospitals' analysis confirms previous results of direct effect of Readiness, Safety, and Empathy on improvement. The coefficient of direct effect is 0.127 of the total effect of 0.186. Hence, the indirect effect is represented by a coefficient of 0.059 which is larger than that for public hospitals of 0.02. This indicates that Efficiency only plays a partial role as a mediator in the relationship of these variables on improvement in the case of private hospitals.

**Table 6:** The mediation role analysis.

**Independent** **variable**	**Dep. variable**	**Public hospitals**		**Private hospitals**	
		**β**	***t*-statistics**	**β**	***t*-statistics**
Efficiency (Mediator)	α	0.881^***^	3.874	0.314^*^	1.658
	RSE	0.242^***^	8.719	0.279^***^	15.331
	*R* ^2^	0.610			
	F-Statistic	235.027			
	*P*-value	0.000			
Improvement (Direct Effect)					
	α	−0.113	−0.823	−0.111	−1.014
	RSE	0.164^***^	7.407	0.127^***^	7.598
	Efficiency	0.084	1.253	0.213^***^	4.560
	*R* ^2^	0.699		0.696	
	F-Statistic	75.338		170.272	
	*P*-value	0.000		0.000	
Improvement (total effect)					
	α	−0.039	−0.313	−0.044	
	RSE	0.184^***^	12.159	0.186^***^	
	*R* ^2^	0.691		0.653	
	F-Statistic	147.829		282.467	
	*P*-value	0.000		0.000	

***,**,* represent significance at 1, 5, and 10 percent, respectively.

## Discussion

Syria is a developing country that face enormous challenges. Suffering from low resources, low service quality, shortage in protective equipment for the medical and nursing staff (Derida,20) due to war conditions. The outbreak of COVID-19 aggravated the already difficult situation and show the fragility of the healthcare system.

In the study, we evaluate the perceived service quality based on hospital type, public and private, using five HEALTHQUAL dimensions. We find that service quality in private hospitals is perceived better that in public hospitals. However, neither type of hospitals scores exceptionally high in any of the examined HEALTHQUAL dimensions. We argue that crowdedness environment, medical staff availability and their salaries, pricing policies as well as the health insurance system, are to blame for such low perceived quality.

The investigation of the impact of the examined four dimensions of HEALTHQUAL on improvement suggests that all these variables load significantly on improvement and contribute toward the perceived improvement of private hospitals' healthcare services. We also find that Efficiency plays a mediator role in the relationship between Improvement and Readiness, Safety, and Empathy. However, Efficiency fails to affect Improvement at Syrian public hospitals.

The results of this study provide valuable insights to researchers, policymakers, managers, and patients. The novelty of this study lies in that it compares the quality of healthcare services between public and private hospitals in the special context of COVID-19 outbreak period and in a healthcare system that was on the edge of collapse due to war conditions. Policymakers and managers are increasingly interested in measuring and improving the service quality since healthcare service quality is one of the main factors that affect hospital choice. It is also of great importance for patients who select the hospital they visit on the basis of fellow recommendations. Hence, we present some practical implications for improving service quality for both private and public hospitals. Many of these prominent bottleneck inefficiencies of response to Pandemics challenge were also noted in an array of comparable health systems sharing some historical legacy with Syria's one in medical care provision and financing (56, 57).

This study rings the alarm bell that patients are unsatisfied with healthcare services provided by public hospitals. Surprisingly, public hospitals failed at all HEALTHQUAL dimensions. Policymakers should address patients concerns regarding service quality at public hospitals. It is suggested that the almost free-of-charge policy applicable at public policy, with its negative consequences such as crowdedness, is the one to blame for the perceived low service quality. We recommend that policymakers consider introduce changes in pricing policies at public hospitals to allow reasonable fees. The fees collected should provide improvements in closing the gap between public and private hospitals' service quality levels. In addition, public hospital patients, who have longer waiting times than a pre-determined threshold, should be directed to private hospitals where their fees for hospital services should be covered by government. Following such suggestions, if the crowdedness of public hospitals decreases, it is believed that medical staff at public hospitals will provide more patient-centered care interventions, develop a consistent positive patient safety culture across the hospital. Furthermore, policymakers and managers of public hospitals should develop a performance evaluation system that encourage receiving feedbacks for patients. These may lead to an improvement in the service quality of public hospitals (35). In the long-term perspective quality of large public hospitals affects the entire fiscal sustainability of the health system (58, 59).

Surprisingly and despite that private hospitals outperformed their public counterparts, private hospital performance at all dimensions as far below expectations. It is unexplained that private hospitals, while charging high fees for treatment, are not scoring quite high on all HEALTHQUAL dimensions. Private hospitals should pay more attention to HEALTHQUAL dimensions and particularly to improvement dimension by providing follow-up service to patients and provide patients with the best possible treatment.

The health insurance system is one of the main causes of such low perceived healthcare quality. The refusal of insurance companies to cover COVID-19 treatment meant that those who are diagnosed with the disease do not receive full treatment and they are discharged before their full recovery and before they feel their health conditions are improved. The expected result is that they are dissatisfied with this service quality. Such findings indicate lack of adaptive capability by the health system affected with a large-scale epidemic which has been documented in other Mediterranean and Asian health systems (60).

Finally, healthcare authorities should recognize that increase the awareness of public of COVID-19 and other pandemics is a priority that will have many advantages. On the one hand, it will reduce the transmission of disease and consequently the number of patients and deaths. On the other hand, such awareness will make hospitals care more about hygiene issues and consequently increase the perceived healthcare quality.

## Study limitations

There were a number of limitations in the study. First, the research is expected to have the majority of respondents from Damascus, the capital of Syria. We fear that our results may not reflect the service quality perceptions in hospitals from all around Syria. Even though the results confirmed the results of previous studies conducted in other countries, future research that includes country-specific hospitals or healthcare service quality models should also be conducted. Second, the study implemented an e-questionnaire, which definitely bias our sample toward young and internet users. In future research, it is advised to increase higher age representation through manual distribution of questionnaires to enable researchers to generalize their results. Third, other healthcare quality dimensions could be used in order to double check the results obtained from HEALTHQUAL measure.

## Data availability statement

The raw data supporting the conclusions of this article will be made available by the authors, without undue reservation.

## Author contributions

LA designed the questionnaire and wrote the literature review. SM wrote the conclusion and limitations. MJ revised the draft and developed the discussion. All authors contributed to the article and approved the submitted version.

## Conflict of interest

The authors declare that the research was conducted in the absence of any commercial or financial relationships that could be construed as a potential conflict of interest.

## Publisher's note

All claims expressed in this article are solely those of the authors and do not necessarily represent those of their affiliated organizations, or those of the publisher, the editors and the reviewers. Any product that may be evaluated in this article, or claim that may be made by its manufacturer, is not guaranteed or endorsed by the publisher.
